# GIS Modeling of Air Toxics Releases from TRI-Reporting and Non-TRI-Reporting Facilities: Impacts for Environmental Justice

**DOI:** 10.1289/ehp.7066

**Published:** 2004-09-13

**Authors:** Dana C. Dolinoy, Marie Lynn Miranda

**Affiliations:** ^1^Integrated Toxicology Program and; ^2^Children’s Environmental Health Initiative, Nicholas School of the Environment and Earth Sciences, Duke University, Durham, North Carolina, USA

**Keywords:** air dispersion modeling, air toxics, environmental justice, geographic information systems (GIS), Toxics Release Inventory (TRI)

## Abstract

The Toxics Release Inventory (TRI) requires facilities with 10 or more full-time employees that process > 25,000 pounds in aggregate or use > 10,000 pounds of any one TRI chemical to report releases annually. However, little is known about releases from non-TRI-reporting facilities, nor has attention been given to the very localized equity impacts associated with air toxics releases. Using geographic information systems and industrial source complex dispersion modeling, we developed methods for characterizing air releases from TRI-reporting as well as non-TRI-reporting facilities at four levels of geographic resolution. We characterized the spatial distribution and concentration of air releases from one representative industry in Durham County, North Carolina (USA). Inclusive modeling of all facilities rather than modeling of TRI sites alone significantly alters the magnitude and spatial distribution of modeled air concentrations. Modeling exposure receptors at more refined levels of geographic resolution reveals localized, neighborhood-level exposure hot spots that are not apparent at coarser geographic scales. Multivariate analysis indicates that inclusive facility modeling at fine levels of geographic resolution reveals exposure disparities by income and race. These new methods significantly enhance the ability to model air toxics, perform equity analysis, and clarify conflicts in the literature regarding environmental justice findings. This work has substantial implications for how to structure TRI reporting requirements, as well as methods and types of analysis that will successfully elucidate the spatial distribution of exposure potentials across geographic, income, and racial lines.

Temporal, spatial, and other special circumstances can result in disproportionate environmental exposures and may play a role in differential health outcomes. For example, numerous studies highlight the disparate impact of asthma and allergies on specific subsets of the population, including minorities and poor families, as well as people living in urban environments (Oliveti et al. 1996; Weitzman et al. 1990; Wissow et al. 1988). In addition, people of color are more likely to live in areas that fail to meet national ambient air quality standards. In 1990, 57% of whites, 65% of African Americans, and 80% of Hispanics lived in counties that exceeded one of the federal criteria air pollutant standards (Wernette and Nieves 1992). In addition, 12% of whites, 20% of African Americans, and 31% of Hispanics lived in counties that failed to meet the federal standard for three or more criteria air pollutants (Wernette and Nieves 1992).

The Toxics Release Inventory (TRI), created by the Emergency Planning and Community Right-to-Know Act (EPCRA 1986), requires manufacturing facilities with ≥ 10 full-time employees that process > 25,000 lb in aggregate or use > 10,000 lb of any one of the 650 TRI chemicals to report their releases and waste management strategies annually to the U.S. Environmental Protection Agency (U.S. EPA 2001a). In January 2001, the U.S. EPA lowered the reporting requirement for lead and lead compounds to 100 lb (U.S. EPA 2001b). Reporting requirements cover emissions from routine processing and/or accidental releases as well as chemicals managed as waste from businesses categorized in Standard Industrial Classification (SIC) codes 10, 12, 20–39, 49, and 51, including metal and coal mining, printing, chemical, paper, electronic, plastics, refining, metal, and other industries. Facilities that fail to report annual releases by 1 July each year are subject to fines of up to $27,500/day (U.S. EPA 2001c).

The purpose of EPCRA is 2-fold. First, it seeks to provide citizens with information on chemical releases and waste management activities in and around their neighborhood, thereby empowering them to hold companies accountable for their emissions (U.S. EPA 2001c). Second, it attempts to provide government agencies with data for research and policy development (U.S. EPA 2001c). Although it is an important advance in helping communities understand the local air toxics load, the TRI program suffers from at least three weaknesses. First, minimum reporting requirements do not require smaller industrial facilities to report. Theoretically, cumulative effects of smaller non-TRI-reporting facilities might outweigh the individual effect of larger (but fewer) TRI-reporting facilities. Second, the U.S. EPA’s TRI database (as well as TRI data organized and maintained by environmental interest groups) does not address environmental fate and transport of industry emissions using modeling and other analytical techniques. The characteristics of pollutant concentration distributions depend on a variety of factors, including media emitted, physical properties of the chemical, wind direction and speed, meteorologic conditions, and stack height. Finally, by reporting emissions at the county level, the TRI database fails to capture important highly localized equity impacts.

The analysis presented here develops a methodology to characterize releases inclusively by incorporating emissions from smaller, non-TRI-reporting facilities. In addition, the project is carried out at four geographic levels of resolution (ZIP code, census tract, census block group, and census block) to assess the importance of geographic resolution in analyzing air toxics emissions. In this article we model pollutant concentrations using dispersion modeling and geographic information systems (GIS) analysis and hypothesize that inclusive modeling of releases from TRI-reporting and smaller non-TRI-reporting facilities at finer levels of geographic resolution will change the distribution of exposure potential for people living in a given area and subsequently improve the quality of equity analysis.

## Materials and Methods

### Study area.

**Figure 1:**
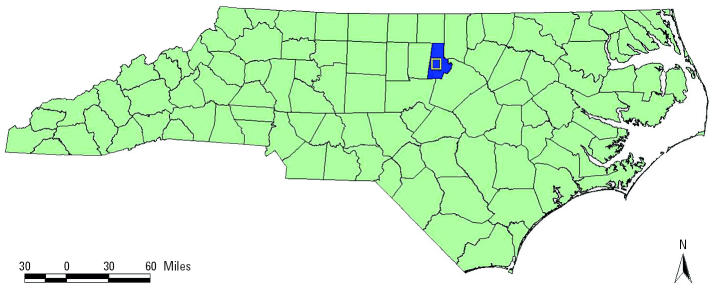
Durham county, North Carolina (USA).

**Figure 2:**
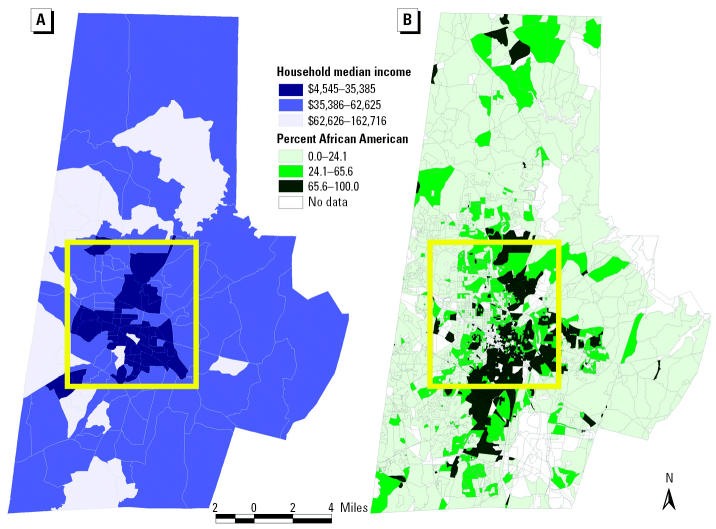
Two demographic variables for Durham County: (*A*) median household income and (*B*) percent African American.

The analysis focuses on Durham County, North Carolina (USA), which represents a broad range of values across demographic, socioeconomic, and social indicators. Table 1 shows selected demographics for Durham County from 2000 Census data (U.S. Census Bureau 2003). Forty percent of Durham County residents are African American, and almost 8% are Hispanic (U.S. Census Bureau 2003). In addition, approximately 10% of residents are living in poverty, and almost 46% reside in rental properties (U.S. Census Bureau 2003). Compared with Durham County as a whole, the State of North Carolina, and the United States, central Durham has a higher percentage of minorities, higher percentage of families in poverty, higher percentage of children < 6 years of age in poverty, lower median household income, and higher percentage of renter-occupied housing (U.S. Census Bureau 2003). Figure 1 highlights the location of Durham County within North Carolina. The yellow box represents central Durham. Figure 2 depicts two demographic variables for Durham County (percent African American by 2000 census block and median household income by 2000 census block group). Note that areas within the yellow box (central Durham) are characterized with a higher percentage of African Americans and lower household median income.

### Demographic data.

**Figure 3:**
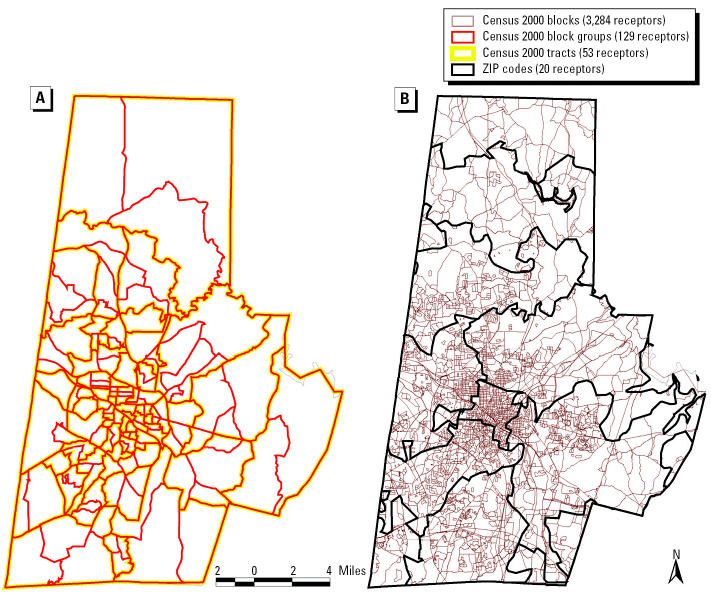
Boundaries for Durham County for (*A*) census tracts and census block groups and (*B*) census blocks and ZIP codes.

This project uses year 2000 demographic data from the U.S. Census Bureau. Census demographic information is available in four different geographic scales: ZIP code tabulation areas (ZCTAs), tracts, block groups, and blocks. ZIP codes and tracts designate the largest geographic areas. The most detailed and focused information is contained in blocks. Blocks are also combined into block groups, an intermediate category. Durham County contains 20 ZIP codes, 53 tracts, 129 census block groups, and 3,284 census blocks. Figure 3 depicts ZIP code, tract, block group, and block boundaries for Durham County.

### Facility data.

Year 2000 TRI data were extracted from the U.S. EPA’s TRI Explorer (U.S. EPA 2002a) and uploaded into a GIS (ArcView 3.2; Environmental Systems Research Institute, Redlands, CA). TRI facility locations and emissions were geocoded to a base map using latitude and longitude. Facility location was cross-referenced against tax parcel data to ensure accurate geolocation. TRI data from 2000 indicate that North Carolina is home to 874 TRI sites, releasing > 126 million lb of contaminants to the air. Durham County is home to 16 of these sites, releasing > 48,000 lb of contaminants to the air.

**Figure 4:**
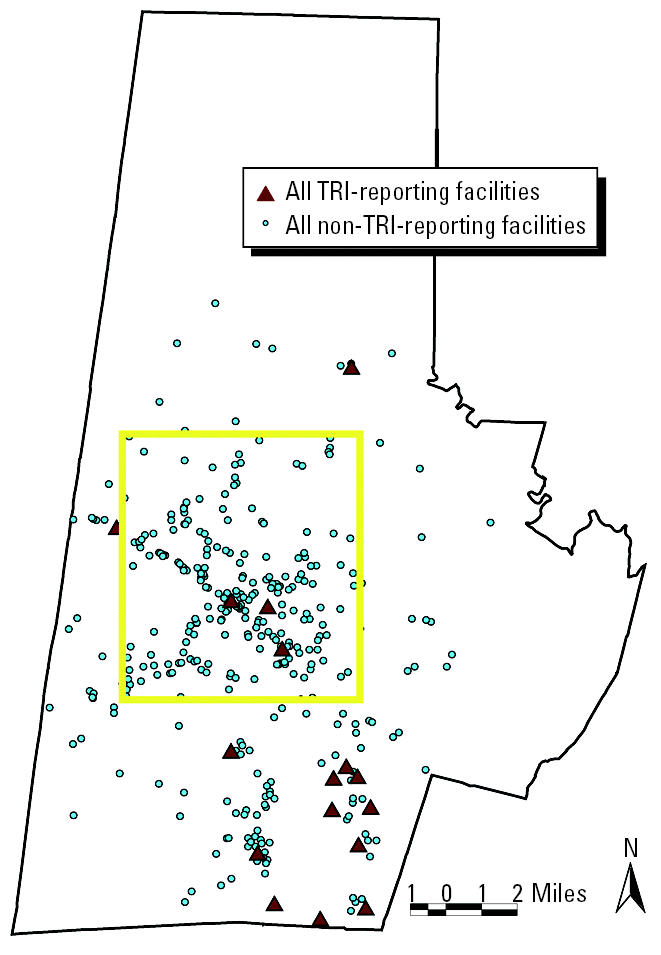
TRI-reporting and non-TRI-reporting facilities in all TRI SIC codes in Durham County. The yellow box represents central Durham.

Nonreporting facilities within TRI-reporting SIC codes were extracted from city marketing directories and imported into the GIS project. Facility locations were address-geocoded to the individual tax parcel unit. City marketing data (infoTYME version 4.1; Polk City Directories, Livonia, MI) contain countywide listings of business names, addresses, contact persons, employee range, and SIC codes. Year 2000 city marketing data (infoTYME Polk City Directories 2000) indicate that Durham County contains > 400 non-TRI-reporting industrial facilities classified in TRI-SIC reporting codes. Figure 4 maps facilities that were required to report to TRI and those in the same SIC codes that were not required to report to TRI in Durham County in 2000. Note that although only three of the TRI-reporting facilities are located in the low-income, predominantly minority communities of central Durham, most of the non-TRI-reporting, smaller facilities are situated in central Durham.

### Selecting a base case.

Ideally, all SIC codes and pollutants subject to TRI guidelines would be evaluated in an aggregate model. However, to facilitate the development of the spatial methods described here, we selected a prototype SIC code and pollutant to model. On the basis of an evaluation of year 2000 air releases from several southern states, we selected a four-digit rather than two-digit SIC code as a base case. This selection criterion better supported the required modeling assumptions that a TRI facility within a defined SIC code releases a similar pollutant profile when compared with other TRI- and non-TRI-reporting facilities in the same SIC code.

**Figure 5:**
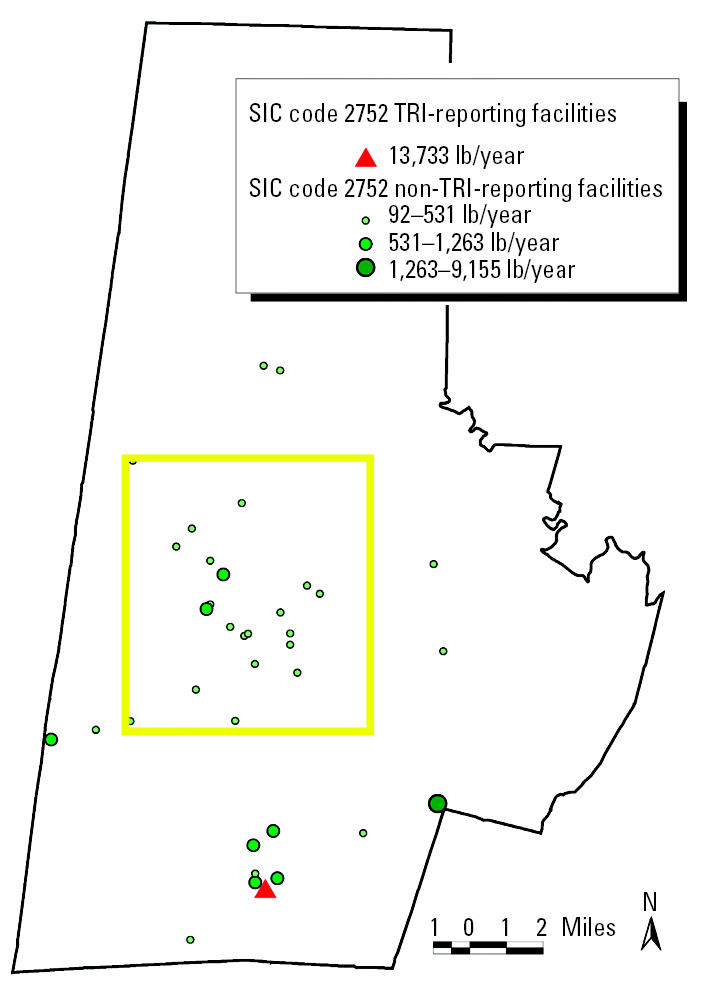
TRI-reporting and non-TRI-reporting facilities in SIC Code 2752 (printing-lithography) and their estimated emissions of certain glycol ethers. The yellow box represents central Durham.

Of the 16 TRI-reporting sites in Durham County, no two facilities were defined within one four-digit SIC code. Therefore, we based the prototype selection on quantity and type of air releases. We chose SIC code 2752, representing commercial lithographic printing, because it released one type of pollutant rather than multiple classes of pollutants. In addition, it ranked second within Durham County for total air releases. Within Durham County, SIC code 2752 contained one TRI-reporting and 36 non-TRI-reporting sites. The TRI-reporting site was located in southern Durham, whereas the 36 non-TRI code 2752 sites were spread throughout the county (Figure 5). The TRI-reporting site emitted “certain glycol ethers” as fugitive (rather than stack) releases to the air.

An evaluation of air emissions in six southern states with manufacturing facilities classified in SIC code 27 revealed wide variability among two-digit SIC codes. However, variability was low when facilities were restricted to four-digit SIC codes. For example, of the 16 facilities in the neighboring state of Virginia with two-digit SIC code 27 releasing TRI chemicals to the air in 2000, three were identified within four-digit SIC code 2752. All three facilities released “certain glycol ethers” or ethylene glycol to the air. In addition, for all three facilities, the overwhelming majority of the releases were identified as fugitive rather than stack.

Glycol ethers represent a bundle of chemicals used in many industries (including printing) as solvents (Environmental Defense 2002). According to 2000 TRI data, the TRI-reporting facility (PBM Graphics) in Durham County released 13,733 lb of “certain glycol ethers” to the air. All releases were classified as fugitive rather than point source. To determine the specific chemical used, we contacted PBM Graphics directly. According to PBM Graphics personnel, the company used ethylene glycol monobutyl ether in 2000. Ethylene glycol monobutyl ether is a nonphotoreactive volatile organic compound used as a solvent during printing processes. It is a suspected cardiovascular, blood, developmental, endocrine, gastrointestinal, kidney, neurologic, and respiratory toxicant [Environmental Defense 2002; New Jersey Department of Health and Senior Services (NJDHSS) 2001]. According to the CalTOX multimedia and multiexposure model, ethylene glycol monobutyl ether exposure potential is primarily through air (rather than settled materials) and induces health effects via inhalation and ingestion [State of California Department of Toxic Substances Control (SCDTSC) 2002]. The half-life of ethylene glycol monobutyl ether is approximately 16 hr in the air. Both the exposure properties and half-life make ethylene glycol monobutyl ether an appropriate pollutant for air dispersion modeling. Although other facilities within SIC code 2752 may emit different subtypes of “certain glycol ethers,” the exposure properties and half-lives of individual compounds do not vary significantly enough to invalidate modeled concentrations.

### Emissions estimation algorithm.

Comparing TRI-reporting with non-TRI-reporting sites requires estimation of emissions from non-TRI-reporting facilities. Ideally, annual averages of production units would be used. However, number of employees can serve as a proxy when production units are not readily known. Therefore, we generated an employee-based emissions algorithm to impute emissions to non-TRI-reporting facilities. The general algorithm for computing the estimated emissions is as follows. Step 1 involves the calculation of a per-employee emissions rate for a chemical of concern (*C*_1_) based on data from all TRI-reporting facilities (*F*_1_, *F*_2_, *F*_3_, … , *F_n_*). In step 2, emissions are imputed to non-TRI-reporting facilities by multiplying the per-employee emissions rate (based on the TRI-reporting facilities) by the number of employees working at each of the non-TRI-reporting facilities.

Using employees instead of production units fails to address how economies of scale might affect production patterns. However, the literature (Dasgupta et al. 2002; Little et al. 1987; U.S. EPA 2001a) indicates that smaller facilities tend to emit more pollution on a per unit of production basis than do larger units, so our imputed data likely underestimate emissions from non-TRI-reporting facilities. Therefore, if significant differences are noted between TRI-reporting models and inclusive TRI-reporting plus non-TRI-reporting sites, under this conservative approach the actual effect can be assumed to be greater.

To impute emissions for the 36 non-TRI-reporting facilities in code 2752, a per-employee emissions rate (based on emissions from PBM Graphics) was calculated. PBM Graphics employed approximately 375 employees in its printing facility in 2000. The reported emissions were 13,733 lb of glycol ethers, delivering an imputed emissions rate of 36.6 lb/employee. As a data check, several SIC code 2752 facilities in surrounding states had similar ratios of emissions per employee. We used city marketing directories (infoTYME Polk City Directories 2000) to ascertain the number of employees at the 36 non-TRI-reporting facilities. Using the per-employee emissions rate and the number of employees, we imputed air emissions for the 36 non-TRI-reporting facilities. Once the base-case methodology is developed, future analysis should include Monte Carlo simulations that vary the per-employee emissions rate across non-TRI-reporting facilities. Figure 5 depicts the 37 facilities in Durham County classified in SIC code 2752 and their corresponding imputed emissions. According to imputed emissions estimates, the 36 non-TRI-reporting facilities emitted a total of 22,156 lb/year.

### ISC dispersion modeling.

Developed by the U.S. EPA (U.S. EPA 2002b), the industrial source complex (ISC) model is one of the most widely used and successful steady-state Gaussian-based air dispersion models. Major assumptions of Gaussian models include *a*) that the rate of plume diffusion is proportional to contaminant concentration, *b*) a constant emissions rate, *c*) a conservative pollutant (no chemical reactions or biodecay), *d*) relatively flat terrain, and *e*) perfect ground reflection. Because of these assumptions, Gaussian models are most appropriate for local applications within 50 km or 2,500 km^2^ (Masters 1998). Gaussian models incorporate two dispersion coefficients based on the standard deviations of the horizontal and vertical Gaussian distributions of the downwind plume dispersion (Masters 1998). The standard deviations or dispersion coefficients increase with distance downwind of the source. In addition to distance, the dispersion coefficients also consider atmospheric stability parameters that address qualitative descriptions of prevailing weather conditions such as season, time of day, and degree of cloud cover. Gaussian model output reports annual average concentration of pollutant for each defined receptor. Receptors are user-defined areas of interest and often include the geographic centroid of ZIP codes or census tracts or points on regular grids spanning a study region.

This analysis used the short-term ISC model, ISCST3 (U.S. EPA 2002b), which captures initial mixing phenomena at the source and is best suited for study areas less than 2,500 km^2^. Containing approximately 751 km^2^, Durham County falls well within this limitation. ISCST3 modeling allows for multiple source and receptor specifications and requires users to input a year’s worth of hourly meteorologic data from the National Weather Service (U.S. EPA 2002c). The modeling period was 1 January 2000 to 31 December 2000. Source emission rates were treated as constant over 1 year by converting the number of pounds released in calendar year 2000 to grams per second.

#### Input requirements.

Within ISCST3, sources may be specified as POINT (stack), AREA (storage piles or irregular shapes), or VOLUME (multiple vents or conveyor belts) types (U.S. EPA 1995). According to TRI Explorer and conversations with personnel from TRI and non-TRI-reporting sites in SIC code 2752, releases from printing lithography facilities are generally fugitive rather than point. The AREA rather than VOLUME type was selected in the ISCST3 model to control for differences in building size. The area of each building footprint (in square meters) was based on the area of PBM Graphics scaled to the number of employees. Furthermore, the AREA subtype is best suited for low-level releases with no plume rise (U.S. EPA 1995). Area emission rates based on the per-employee emissions algorithm were entered into ISCST3 in grams per second meter squared. An average release height of 5 m was specified for each source. Source coordinates were entered as universal transverse mercator (UTM) coordinates.

A year’s worth of hourly meteorologic data were compiled using the PCRAMMET meteorologic preprocessor program (U.S. EPA, Research Triangle Park, NC). Ground-level weather data were obtained from the Raleigh–Durham Airport surface station (approximately 3 miles southwest of Durham County). The anemometer height at the airport is approximately 10.1 m. Mixing height data from the National Climatic Data Center (U.S. EPA 2002c) were obtained for the closest station (Greensboro, NC), located approximately 50 miles west of Durham County. The twice-daily mixing height values were combined with hourly surface data using PCRAMMET to derive hourly interpolated mixing height values.

We defined receptors as the geographic centroid of four modeling units (ZIP code, census tract, census block group, and census block) in Durham County, North Carolina. For each geographic scale, receptor coordinates of the centroids were entered as UTM coordinates. The default receptor elevation of ground level was used. Areas adjacent to Durham County were not specified or analyzed in this study.

To control for pollutant fate and transport characteristics, the ISCST3 model allows users to input pollutant half-life and specify average land terrain across the study area of interest. As an initial exercise, default values for pollutant half-life and landscape terrain were first specified. The ISCST3 default pollutant half-life is 4 hr. However, the half-life of ethylene glycol monobutyl ether is 16 hr. When the default half-life was changed to 16 hr, we observed no significant changes in output results. The default ISCST3 landscape terrain is rural. Because the landscape of Durham County is variable, the models were also specified using an urban terrain. Results did not vary significantly based on rural versus urban specified landscape terrain.

#### Models.

To assess the importance of inclusive modeling of all emitters as well as the importance of geographic resolution, we specified eight ISCST3 models: 1a, TRI-reporting sites alone at ZIP code level (20 receptors); 1b, all emitters at ZIP code level (20 receptors); 2a, TRI-reporting sites alone at tract level (53 receptors); 2b, all emitters at tract level (53 receptors); 3a, TRI-reporting sites alone at block group level (129 receptors); 3b, all emitters at block group level (129 receptors); 4a, TRI-reporting sites alone at block level (3,824 receptors); 4b, all emitters at block level (3,824 receptors).

Each model run generated an average concentration of the pollutant in micrograms per cubic meter in a particular receptor grid for the entire year. Comparing run a with run b (e.g., comparing 1a and 1b) allows for determination of the importance of inclusive modeling—that is, whether including non-TRI-reporting facilities changes exposure potential across different demographic groups. Comparing runs 1–4 explores the importance of geographic resolution in analyzing contaminant distribution across demographic groups. Statistical and spatial analysis of pollutant concentration was based on model output and was not verified by collecting environmental samples.

### Statistical and spatial analysis.

Using spatial and tabular tools within GIS, modeled emissions from the ISC dispersion models were aggregated into spatially referenced data sets and combined with underlying census demographic data. The combined data sets were imported into Microsoft Excel (Excel 2000; Microsoft, Seattle, WA) and STATA 8.0 (Stata Corp., College Station, TX) for statistical analysis.

#### Cumulative distribution functions.

Comparing means (or medians) of modeled concentration, as is the case in multivariate regression analysis, may overemphasize differences near the center of the concentration distribution. Many times, scientists and public health analysts are most concerned with areas of relatively high (or low) exposure risk (i.e., the tails of the distribution). Therefore, we used cumulative distribution functions (CDFs) to address disparate exposure potential across two groups. For illustrative purposes, the *y*-axis of a CDF curve represents the percentage of the population (from 0 to 100), and the *x*-axis represents exposure potential (e.g., interpolated monitoring site data or modeled concentration levels) (Lopez 2002).

We estimated the CDFs using techniques reported in Waller et al. (1999). CDFs were created for all four levels of geographic resolution (ZIP code, census tract, census block group, and census block), although only data for census block, the finest level of resolution, are presented here. Population data for each subpopulation of interest for each geographic level of resolution were determined using 2000 census data and GIS. Exposure values were assigned to each unit based on modeled concentration values for the corresponding geographic level of resolution.

#### Multivariate statistical analysis.

To access multiple demographic variables at once, multivariate statistical analysis was performed with STATA 8.0. All dependent variables were log-transformed to normalize right-skewed data.

#### Kriging.

Using the spatial analyst extension within ArcView version 3.2 GIS software, a set of contour lines representing predicted concentration of glycol ethers for the entire Durham County were developed. Using a built-in geostatistical program with user-defined parameters, ArcView software interpolates lines that represent locations with the same pollutant concentration magnitude. Although kriging the modeled data introduces an additional layer of uncertainty, the smoothed contour lines depict a more easily interpreted array of pollutant concentrations, which is extremely useful for neighborhood-level equity analyses and community outreach.

## Results

The outcome variables of interest from the ISC dispersion modeling data sets are *a*) the annual average concentration (micrograms per cubic meter) of glycol ethers based on modeling of the TRI site alone and *b*) the annual average concentration (micrograms per cubic meter) of glycol ethers based on modeling of the TRI site plus non-TRI-reporting sites. Modeling was conducted at four geographic levels: ZIP code, census tract, census block group, and census block. The annual average concentrations were converted to nanograms per cubic meter to facilitate presentation and log transformation.

Table 2 presents descriptive statistics for the annual average concentration in ZIP codes, census tracts, census block groups, and census blocks. Dispersion modeling data range in concentration from 0.3 ng/m^3^ (TRI site alone, ZIP code level) to 821.05 ng/m^3^ (all sites together, census block level). The inclusion of non-TRI-reporting sites in the model increases the average concentration among ZIP codes from 1.5 to 4.9 ng/m^3^. Likewise, the inclusion of smaller non-TRI-reporting sites in the model increases the average concentration among census tracts from 2.7 to 10.3 ng/m^3^; for census block groups, from 3.0 to 10.2 ng/m^3^; and for census blocks, from 3.9 to 12.1 ng/m^3^.

### Importance of inclusive modeling.

**Figure 6:**
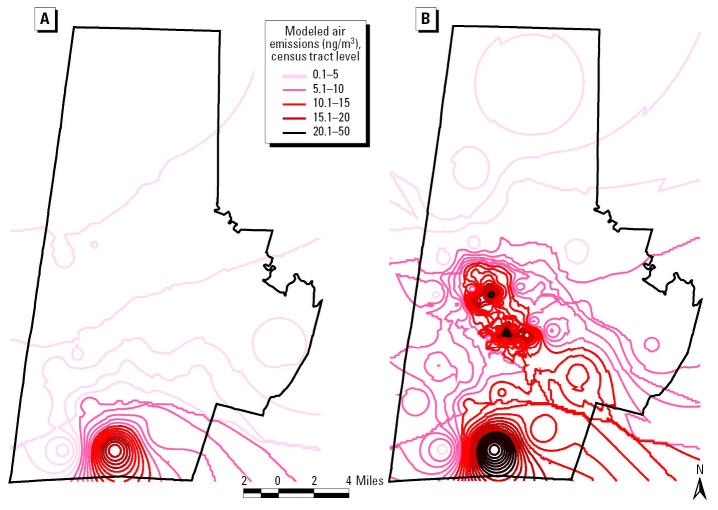
Modeled air emissions (ng/m^3^) of certain glycol ethers for (*A*) TRI-reporting and (*B*) non-TRI-reporting facilities.

As shown in Figure 6, inclusive modeling of all facilities, accomplished by imputing emissions to non-TRI-reporting facilities in the same SIC code, rather than modeling of TRI sites alone, significantly alters the magnitude and spatial distribution of modeled air concentrations. Recall from Figure 2 that areas in southern Durham County have higher household median incomes and relatively low densities of minorities compared with central Durham, as measured by 2000 Census data (U.S. Census Bureau 2003). Note the northward drift of higher concentration contours (the deeper the red color, the higher the modeled concentration) into lower income, predominantly minority communities. Thus, incorporating the non-TRI-reporting facilities provides a substantially different perspective on exposure to contaminants across race and income lines.

For noninclusive modeling at each level of geographic resolution, major impacts occur within a few miles of the TRI site. For inclusive modeling of the TRI-reporting plus non-TRI-reporting facilities at each level of geographic resolution, major impacts are spread throughout Durham County and into adjacent counties (Chatham, Orange, and Wake). The aggregate effects of modeling multiple smaller non-TRI-reporting emissions in central Durham are of the same order of magnitude as the effects of the larger TRI site in southern Durham. Although non-TRI-reporting sites do not significantly affect exposure potential in areas with TRI facilities, they do affect exposure potential in areas at some distance from TRI facilities. This results partly from the size and specific locations of the reporting and non-TRI-reporting 2752 facilities and may not necessarily hold when generalized to other SIC codes.

**Figure 7:**
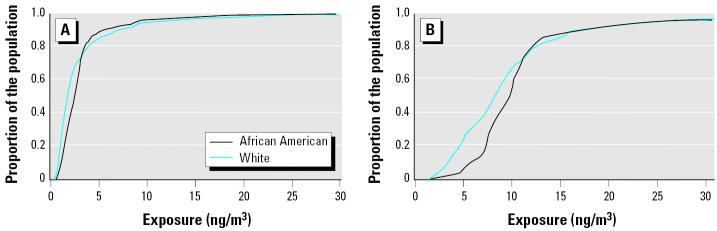
CDF curves of modeled census-block–level exposure for African-American and white subpopulations for (*A*) TRI sites alone and (*B*) all emitters.

Figure 7 depicts the CDF for African-American and white subpopulations modeled at the block level. Figure 7A represents exposure values for noninclusive modeling of air emissions for TRI-reporting facilities only. The CDF curves for African-American and white subpopulations exhibit a narrow gap, indicating that a slightly larger proportion of whites reside in blocks with lower exposure potentials. Figure 7B represents exposure potential values for inclusive modeling of air emissions from all emitters at the block level. Inclusive modeling produces a larger gap between the CDF curves for the African-American and white subpopulations. The increase in exposure disparity moving from noninclusive to inclusive modeling persists at the three coarser levels of geographic resolution (data not shown). However, the increased gap is most apparent at the census block level.

**Figure 8:**
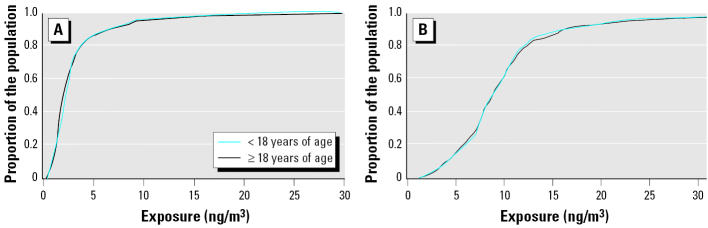
CDF curves of modeled census-block–level exposure for child and adult subpopulations for (*A*) TRI sites alone and (*B*) all emitters.

Figure 8 depicts the CDF curves for comparing adult and nonadult (< 18 years of age) subpopulations. Again, Figure 8A represents exposure values for noninclusive modeling of air emissions for TRI-reporting facilities only. The CDF curves for adults and nonadults overlap, indicating a lack of disparate exposure. Figure 8B represents exposure values for inclusive modeling of air emissions from all emitters. Unlike the CDF for race depicted in Figure 7, potential exposure disparities based on age do not appear to be sensitive to noninclusive versus inclusive modeling. CDFs were also estimated for persons < 5 versus > 5 years of age. Significant differences in exposure potential were not observed based on noninclusive versus inclusive modeling.

### Importance of geographic resolution.

Comparing run 1 (ZIP code receptor) through run 4 (census block receptors) in Table 2 reveals that modeling receptors at a more refined geographic resolution alters the annual average concentrations of glycol ethers for both the TRI models alone and the inclusive all-sites models. The range of concentrations for the census block model is successively greater than the range of concentrations for the coarser geographic scale models. Because the same emissions are being spread over successively smaller areas, the wider range of concentrations at finer geographic scales is an intuitive result.

**Figure 9:**
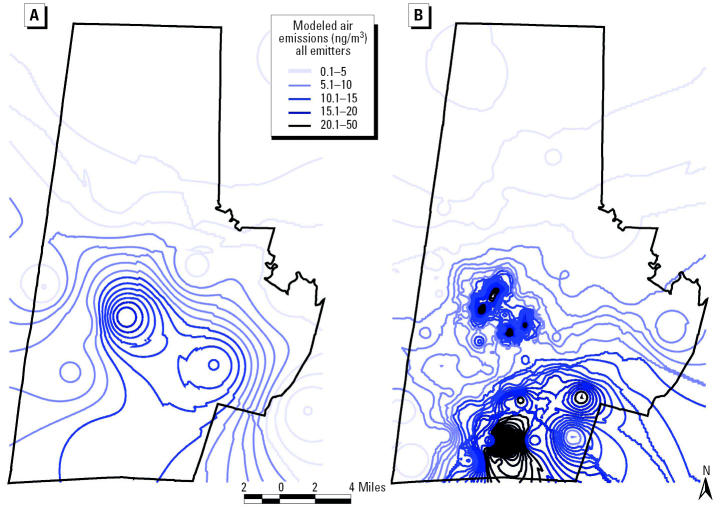
Modeled inclusive air emissions (ng/m^3^) of certain glycol ethers for all facilities at (*A*) the census tract and (*B*) the census block group level.

Figure 9 depicts kriging results with two contour maps representing annual average concentration of glycol ethers for Durham County, North Carolina (the deeper the blue color, the higher the modeled concentration). Both maps represent inclusive modeling of all emitters. However, Figure 9A depicts contours based on modeling sources and receptors at the coarser ZIP code level of geographic resolution, whereas Figure 9B represents modeling at the finer census block group level. As shown in Figure 9, modeling exposure receptors at finer geographic levels of resolution (i.e., census block group rather than ZIP code) reveals localized, neighborhood-level exposure hot spots that are not apparent at coarser geographic scales—note in particular the high concentration contours that appear in central Durham under this alternative modeling approach. Modeling finer geographic levels of resolution provides a substantially different perspective on exposure to contaminants across race and income lines. Unlike the contours at ZIP code level, the contours at census block group level highlight areas in central Durham, characterized by a higher percentage of minorities and a lower median household income, as potential hot spots for exposure.

To better summarize and assess whether modeling of air emissions at varying levels of geographic resolution affects the distribution of exposure potential, we performed multivariate statistical analysis on the relationship between concentration and race and income. We focus specifically on these two variables because of their ubiquitous use in equity analysis. Dependent variables of interest were the modeled concentrations at the four geographic levels of resolution (ZIP code, census tract, census block group, and census block).

Tables 3 and 4 summarize regression results across geographic scale. In Table 3, moving from top to bottom indicates stepping from coarser (ZIP code) to finer (census block) levels of geographic resolution. A positive sign indicates a positive coefficient on the regression coefficient, and S indicates significance at the 0.05 level. Likewise, a negative sign indicates a negative coefficient on the regression coefficient, and NS indicates lack of significance at the 0.05 level. Moving from top to bottom, both the income and minority variables become significant and of the expected sign. These results highlight the importance of spatial resolution in conducting equity analysis. Table 4 presents more detailed regression results from multivariate statistical analysis. Comparing the ZIP code with block models explores the importance of geographic resolution in analyzing contaminant distribution. The census block model, representing inclusive modeling of all emitting sites at a very refined geographic scale, indicates exposure potential disparities across both income and race.

Additional multivariate analyses including the relationships between modeled concentration and percent children, percent vacant housing, and percent receiving public assistance did not reveal any statistically significant trends (results not shown). In addition, percent minority appears to be the most relevant “race” variable based on the modeled data, and median household income appears to be the most important “income” variable based on the modeled data (results not shown).

## Discussion and Conclusion

Previous air toxics and TRI studies have taken advantage of advances in spatial and statistical mapping software to assess how geographic levels of resolution affect environmental justice conclusions. Summarizing many existing geographic-based air toxics studies, Lopez (2002) explains that conclusions often differ depending on the geographic unit of analysis. For example, “micro-area” studies that observe areas with and without facilities conclude that race is not a significant predictor for site but that income may play a role. On the other hand, “meso-area” studies that expand the area of interest to include blocks adjacent to facilities often conclude that race is an important predictor for siting but income is not. Furthermore, results from “macro-level” studies that compare counties with other counties or states with other states have correlated industrial facility siting with large percentages of minorities and persons in poverty. However, the results may be confounded by urban/rural status and other trends. Other traditional environmental justice analysis of industrial siting has focused on the location of facilities and not on concentration distributions and subsequent exposure potential (Morello-Frosch et al. 2002).

Our study attempts to clarify conflicts in the literature regarding facility siting, exposure potential, and equity by developing methods for inclusive modeling of releases at fine levels of geographic resolution. Although the results described here are specific to emissions of glycol ethers from printing/lithography sites in Durham County in 2000, the method developed is relevant across time, space, and industries. This is one of the first studies to develop methods for characterizing and mapping releases from smaller, non-TRI-reporting facilities. The study methodology further characterizes pollutant distribution, fate, and transport by incorporating atmospheric dispersion modeling. The use of GIS as a platform for data storage, statistical analysis, and kriging remains an important cornerstone for conducting spatially based environmental justice research. In addition, although average and maximum annual average concentrations of the pollutant (Table 2) do not approach the noncancer levels of concern set by the California EPA (700,000 ng/m^3^) and the U.S. EPA (20,000 ng/m^3^) (Environmental Defense 2002), the methods developed and presented here represent an innovative prototype for contaminant analysis. A full characterization of exposure potential would take into consideration releases from other sources and adjacent counties.

In a recent article, Maantay (2002) attributes the failure of previous studies to address small polluters such as automobile repair shops to the lack of standardized and publicly available data sets on small polluters. Although there are several caveats to using employees to estimate facility emissions, we believe that the approach offers a sound and creative solution for addressing these data limitations. However, future studies adopting this mechanism should perform some quality assurance techniques to ensure that number of employees reflects a representative proxy to production units and/or pollutant emissions.

The results indicate that the inclusive modeling of all facilities significantly alters the magnitude and spatial distribution of modeled air concentrations (Figure 6). Modeling all sites together rather than modeling TRI sites alone increases the magnitude of modeled concentrations—especially in areas with no TRI facilities. The red concentration contours depicted on the inclusive map are spatially correlated to high-minority and low-income neighborhoods presented in Figure 2. The same correlation is not observed for the TRI-reporting contours. In addition, the CDF curves indicate that inclusive facility modeling at fine levels of geographic resolution results in exposure disparities across race but not age (Figures 7 and 8).

As described above, a significant body of literature exists comparing varying levels of geographic resolution with different exposure potential outcomes (Glickman 2004; Lopez 2002; Maantay 2002; Sheppard et al. 1999). Intuitively, it makes sense that the finer the geographic resolution, the higher the predicted exposure concentration. In this analysis we attempted to build upon these studies by showing that the choice of geographic resolution significantly affects both the significance and trend of multivariate statistical analysis of underlying demographics. In our study, concentration gradients are substantially influenced by the resolution of the model, indicating that receptor choice is a significant modeling parameter and that localized equity impacts may be best represented at the block level.

These new methods significantly enhance the ability to model air toxics and perform equity analysis. They also clarify conflicts in the literature regarding environmental justice findings. In modeling air toxics, both the fate and transport literature and the mechanistic literature indicate that modeling inclusively at a refined geographic scale makes biologic sense. From a policy standpoint, then, it becomes critical to understand how reporting requirements and the design of spatial analyses can shape conclusions. This work, as it moves forward, will also have much to say about which facilities should be required to report to TRI, as well as how much reliability we can place on current TRI data. The 2001 drop in the reporting threshold for lead to 100 lb, which resulted in 251 additional facilities (> 5-fold increase) reporting lead processing in North Carolina, is one example of the impact reporting requirements have on what is known about emissions from local facilities. Future analysis will consider multiple contaminant exposures from multiple industries and explore the use of toxic equivalency factors to better analyze underlying justice concerns and exposure potential.

## Figures and Tables

**Table 1:** Demographics of central Durham, Durham County, State of North Carolina, and United States.

Region	Population (*n*)	Hispanic (%)	African American (%)	Families in poverty (%)	< 6 Years of age in poverty (%)	Median household income	Renter occupied (%)
Central Durham	57,690	12.5	60.5	22.2	37.8	$36,368	64.8
Durham County	223,314	7.6	39.5	9.8	19.9	$43,337	45.7
North Carolina	8,049,313	4.7	21.6	9.0	17.8	$39,184	30.6
United States	281,421,906	12.5	12.3	9.2	18.1	$41,994	33.8

Source: U.S. Census 2000 (U.S. Census Bureau 2003).

**Table 2:** Year 2000 annual average concentration of ethylene monobutyl ether (ng/m^3^) for Durham County.

Geographic resolution	No. of receptors	Model run	Mean	Median	Maximum	Minimum	SD
ZIP code	20	1a: TRI alone	1.5	0.9	5.4	0.3	1.31
		1b: all emitters	4.9	2.8	17.9	0.8	4.4
Census tract	53	2a: TRI alone	2.7	2.1	19.8	0.6	2.8
		2b: all emitters	10.3	9.0	46.4	2.2	7.5
Census block	129	3a: TRI alone	3.0	2.2	28.2	0.5	3.3
group		3b: all emitters	10.2	9.1	49.4	1.6	7.0
Census block	3,824	4a: TRI alone	3.9	2.2	799.2	0.4	19.6
		4b: all emitters	12.1	8.7	821.1	1.2	27.3

**Table 3:** Trends from the multivariate statistical analysis.

Geographic resolution	Inclusive modeling
ZIP code	Minority: +/S
	Income: +/S
Census tract	Minority: +/NS
	Income: −/~S
Census block group	Minority: +/S
	Income: −/NS
Census block	Minority: +/S
	Income: −/S

Abbreviations: +, positive β-coefficient, positively proportional to pollutant concentration; −, negative β-coefficient, inversely proportional to pollutant concentration; NS, lack of significance at 0.05 level; S, significant at the 0.05 level; ~S, significant at the 0.10 level.

**Table 4:** Inclusive modeling regression results at four levels of geographic resolution (*p*-value) from multi-variate statistical analysis.

	ZIP code coefficient	Tract coefficient	Block group coefficient	Block coefficient
Constant	−1.018	2.306	2.066	2.0245
Percent minority	0.0382 (0.0001)*	0.360 (0.212)	0.519 (0.011)*	0.622 (0.0001)*
Household median income	0.0000193 (0.022)*	−7.89 × 10^−6^ (0.081)	−4.03 × 10^−6^ (0.153)	−3.55 × 10^−6^ (0.0001)*
Adjusted *R*^2^	0.50	0.15	0.13	0.11

* Significant at the 0.05 level.
